# Effect of a short-term HAART on SIV load in macaque tissues is dependent on time of initiation and antiviral diffusion

**DOI:** 10.1186/1742-4690-7-78

**Published:** 2010-09-26

**Authors:** Olivier Bourry, Abdelkrim Mannioui, Pierre Sellier, Camille Roucairol, Lucie Durand-Gasselin, Nathalie Dereuddre-Bosquet, Henri Benech, Pierre Roques, Roger Le Grand

**Affiliations:** 1 CEA, Division of Immuno-Virology, DSV/iMETI, Fontenay-aux-Roses, France; 2 Assistance Publique-Hôpitaux de Paris, Hôpital Lariboisière, 2 rue Ambroise Paré, 75010 Paris, France; 3 CEA, Service de Pharmacologie et d'Immunoanalyse, DSV/iBiTecS, CEA/Saclay, 91191Gif-sur-Yvette, France; 4 Université Paris-Sud 11, UMR E01, Orsay, France; 5 Inserm U625, Rennes, France

## Abstract

**Background:**

HIV reservoirs are rapidly established after infection, and the effect of HAART initiated very early during acute infection on HIV reservoirs remains poorly documented, particularly in tissue known to actively replicate the virus. In this context, we used the model of experimental infection of macaques with pathogenic SIV to assess in different tissues: (i) the effect of a short term HAART initiated at different stages during acute infection on viral dissemination and replication, and (ii) the local concentration of antiviral drugs.

**Results:**

Here, we show that early treatment with AZT/3TC/IDV initiated either within 4 hours after intravenous infection of macaques with SIVmac251 (as a post exposure prophylaxis) or before viremia peak (7 days post-infection [pi]), had a strong impact on SIV production and dissemination in all tissues but did not prevent infection. When treatment was initiated after the viremia peak (14 days pi) or during early chronic infection (150 days pi), significant viral replication persists in the peripheral lymph nodes and the spleen of treated macaques despite a strong effect of treatment on viremia and gut associated lymphoid tissues. In these animals, the level of virus persistence in tissues was inversely correlated with local concentrations of 3TC: high concentrations of 3TC were measured in the gut whereas low concentrations were observed in the secondary lymphoid tissues. IDV, like 3TC, showed much higher concentration in the colon than in the spleen. AZT concentration was below the quantification threshold in all tissues studied.

**Conclusions:**

Our results suggest that limited antiviral drug diffusion in secondary lymphoid tissues may allow persistent viral replication in these tissues and could represent an obstacle to HIV prevention and eradication.

## Background

The acute phase of human or simian immunodeficiency virus (HIV/SIV) infections is decisive as it is characterized by a quick and strong decrease of T CD4+ memory cells, particularly in the gut associated lymphoid tissue (GALT), and a rapid spread of the virus in all lymphoid tissues [1,2]. In a recent study, we have shown that during acute SIV infection of macaques, the kinetics of viral dissemination and replication differ between the different lymphoid tissues. Following the peak of viremia, viral DNA and RNA persist at high levels in the secondary lymphoid tissues (spleen and lymph nodes), whereas they rapidly decrease in the blood and the gut [3]. Therefore, this study reinforces the need to explore not only the blood, but also the different lymphoid tissues when assessing strategies aimed at reducing SIV/HIV reservoirs.

HAART initiated very early during infection could prevent the loss of CD4+ T cells from the gut and may delay the onset of the disease [4]. While HIV reservoirs are also set up during acute infection, few studies have focused on the effect of early HAART on tissue viral replication and reservoirs.

Because of the difficulty to obtain tissue samples from HIV infected patients, the macaque models are particularly useful for the exploration of viral dissemination and replication in the different body compartments, especially during the early phases of infection [5]. In the present study, we explored the effect of a short term HAART initiated at different stages during SIV acute infection on the viral burden in the main lymphoid tissues, including the gut. Different quantitative approaches were used, including total SIV-DNA to evaluate viral dissemination and SIV-RNA to assess viral replication and production. As 2LTR SIV-DNA circles have been suggested to accumulate in recently infected target cells [6], we also explored the value of their quantification as an additional method to assess the impact of treatment on viral dissemination.

We show that the Zidovudine (AZT)/Lamivudine (3TC) and Indinavir (IDV) combination could efficiently reduce viral dissemination and replication in all tissues when treatment was initiated before the peak of viremia. Surprisingly, when the same treatment was started after the viremia peak, the effect of treatment was stronger in the gut than in the secondary lymphoid tissues; this is likely due to the very heterogeneous tissue diffusion of several of the antiretroviral drugs.

## Results

### A short term HAART initiated during early chronic SIV infection reduces plasma viral load but has a weak effect on secondary lymphoid tissues

In a first step, to validate our HAART treatment in the SIV macaque model, we assessed the effect of the AZT/3TC/IDV combination in chronically infected animals. Six macaques infected with SIVmac251 were treated with AZT/3TC/IDV twice a day after viral set point (from day 150 pi). To determine the kinetics of viral load decrease in different tissues, three of these animals were killed at day 14 (chronic HAART 14d) and the other three at day 28 (chronic HAART 28d) after the onset of treatment. Three untreated animals at the same stage of infection were used as controls (chronic untreated). Among the HAART treated animals, one (#20929) had undetectable PVL at the initiation of therapy (day 0), but was nevertheless included in the analysis since cell-associated viral load (CVL) in PBMC evolved within the same range of the other treated macaques.

**Figure 1:**
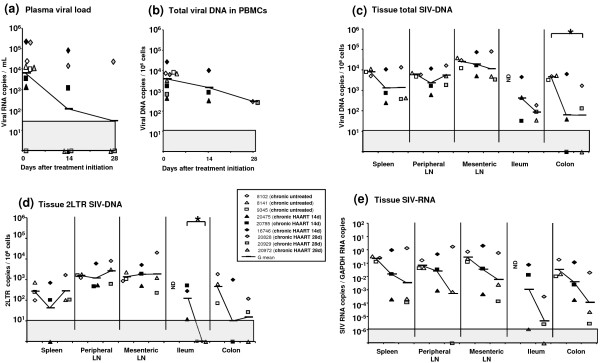
**Viral dynamics in SIV-infected macaques receiving a short-term HAART during early chronic infection**. Among 9 SIV infected macaques at the set point of infection (150 days pi), 3 animals were untreated and killed (chronic untreated: open symbols), 3 animals received a 14 day long (chronic HAART 14d: black symbols) or a 28 day long (chronic HAART 28d: grey symbols) AZT/3TC/IDV treatment and were killed at the end of treatment. In the blood, the treatment induced a decrease of the plasma viral load (**a**), but a weak impact on the cell associated viral load (**b**). In tissue, total SIV DNA and 2LTR circles were almost constant in spleen and lymph nodes but reduced in gut (**c, d**). The treatment reduced the SIV RNA level in all tissues, with a slightly more important decrease in the gut than in the spleen or LN (**e**). *: indicated a significant difference (p < 0.05) using a Mann-Whitney test. ND: not determined due to the unavailability of ileum samples for placebo animals, LN: lymph node, G mean: geometric mean. The grey area indicates the quantitative threshold of our qRT-PCR and qPCR assays.

We confirmed that in macaques chronically infected with SIV, four weeks of HAART result in a decrease of plasma viral load (PVL) similar to that observed in HIV-infected patients receiving similar treatment [7]. As early as day 14 of treatment, the PVL was reduced by 1.7 × log10 in geometric (G) mean, and three of these animals had decreased PVL below detection threshold (60 vRNA copies/ml) (Figure 1a). After 28 days of therapy, only one macaque (#20828) had persistent viremia which was slightly reduced when compared to day 0 (-0.9 × log10).

We then explored viral dissemination and replication in PBMC, spleen, lymph nodes (LN) and the gut to assess whether the decrease in plasma viral load reflects similar dynamics in the other tissues. As previously observed in patients infected with HIV, SIV-DNA in PBMC was only slightly reduced by treatment, with G mean reductions of 0.4 × log10 and 1.1 × log10 after 14 and 28 days of treatment, respectively (Figure 1b). Interestingly, the level of SIV-DNA was not significantly reduced in the lymph nodes and spleen of treated animals (Figure 1c). We surmised that the limited effect of treatment in these tissues could be explained by the limited diffusion of antivirals in these compartments and/or the inefficiency of the drug on long-lived infected cells. In contrast, treatment had a strong effect in the digestive tract with a G mean reduction of 1.8 × log10 in colon (p = 0.049 at day 28). This tissue has predominantly short-lived CD4+ T cells facilitating the effect of HAART [8]. Also, the antivirals may diffuse better in these tissues.

We then measured levels of 2LTR SIV-DNA, as a marker of recently infected cells as previously suggested [6]. In untreated animals, we detected 2LTR SIV-DNA in all the tissues studied. In macaques treated with HAART for 28 days, levels of 2LTR SIV-DNA were significantly reduced (p = 0.04) in the ileum when compared to animals receiving only 14 days of HAART. On the contrary, the antiviral treatment had no effect on the 2LTR SIV-DNA levels in the LN and the spleen. Although the value of 2LTR viral DNA circles is still controversial, our result suggests that treatment probably affects efficiently the tissues with a predominance of new infections (Figure 1d).

Finally, we measured SIV-RNA levels to assess the production of SIV. Under treatment there is a significant (p = 0.03) decrease of SIV-RNA levels if we consider all compartments; but due to the limited number of animals in each group, the effect was not significant for each tissue separately. The strongest effect was observed in the gut, with a G mean decrease of 2.4 × log10 (Figure 1e), confirming the results observed for viral DNA in the GALT. The effect of treatment on SIV-RNA levels was more limited in the LN and spleen (G mean decrease of tissue viral load: 1.8 × log10).

### AZT/3TC/IDV treatment initiated before viremia peak results in partial control of viral dissemination and replication in tissues

When started during early chronic infection, a short term HAART showed a limited and not significant effect in LN and spleen. We then evaluated the effect of this treatment initiated at earlier stages hypothesizing that we might expect a better efficacy of HAART since viral reservoirs are probably not yet fully established.

**Figure 2:**
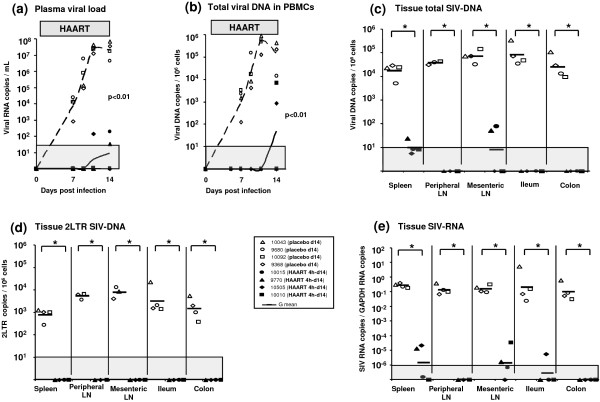
**Viral dynamics in SIV-infected macaques receiving a short-term post exposure HAART**. Four hours after SIV intravenous inoculation, 2 groups of 4 macaques received either the AZT/3TC/IDV combination (HAART 4h-d14) or a placebo (placebo d14), then were killed at the end of treatment (14 days pi). After intravenous infection, post exposure HAART controlled the plasma (**a**) and the cell associated viral load (**b**). In tissues collected 14 days after inoculation the viral dissemination (**c**) and replication (**d, e**) were almost undetectable in HAART 4h-d14 animals (black symbols), whereas placebo d14 animals demonstrated extensive viral propagation (**c**) and replication (**d,e**) (open symbols). *: indicated a significant difference (p < 0.05) using a Mann-Whitney test. LN: lymph node, G mean: geometric mean. The grey area indicates the quantitative threshold of our qRT-PCR and qPCR assays.

In a first experiment, two groups of four SIV-infected animals received AZT/3TC/IDV treatment or a placebo starting 4 h after intravenous inoculation of SIV. Animals were killed at day 14 pi, when viral replication and dissemination were expected to be maximal in placebo treated macaques (Figure 2a-e). Confirming our previously published observations [9], although early treatment could not prevent infection after intravenous transmission, at day 14 pi, PVL and CVL in HAART treated animals remained almost undetectable (Figure 2a-b). Although levels of viral RNA and DNA were close to the quantification threshold, virus remained detectable in spleen and mesenteric lymph nodes, confirming that residual SIV replication and dissemination persisted despite HAART was initiated very early (4h) after intravenous inoculation of SIV.

**Figure 3:**
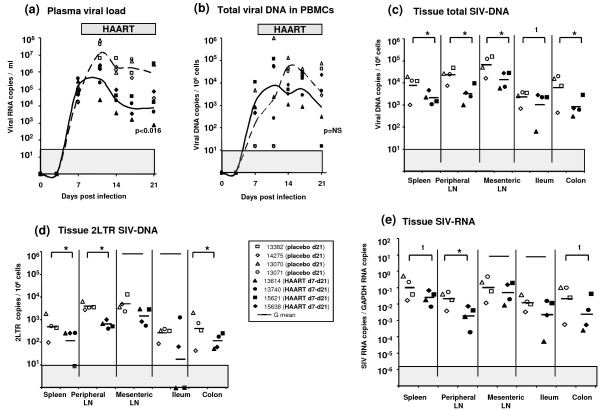
**Viral dynamics in SIV-infected macaques receiving a short-term HAART before the viremia peak**. Seven days after SIV infection, 4 macaques received either a placebo (placebo d21: open symbol) or the AZT/3TC/IDV combination (HAART d7-d21: black symbol) for 14 days and were killed at the end of treatment (21 days pi). Initiation of HAART 7 days after SIV infection conducts to a significant decrease of PVL **(a) **but non-significant reduction of CVL **(b)**. Viral dissemination is also slightly (but significantly) impacted in almost all tissues **(c)**, whereas viral replication is only reduced in the spleen, peripheral LN and colon **(d, e)**. Due to its controller profile, placebo animal #14275 was excluded from statistical analysis. *: indicated a significant difference (p < 0.05) and t a trend (0.05 < p < 0.06) using a Mann-Whitney test. LN: lymph node, G mean: geometric mean. The grey area indicates the quantitative threshold of our qRT-PCR and qPCR assays.

In a second experiment, two groups of four SIV-infected animals received AZT/3TC/IDV treatment or a placebo between 7 and 21 days post-infection and were killed at 21 days pi. In these animals, the effect of antiretroviral treatment was intermediate between what was observed in macaques treated as early as 4 h pi and macaques treated during early chronic infection. Thus, the PVL at day 21 pi was significantly lower in HAART treated animals than in placebo treated macaques (-2 × log10, p = 0.02), but no significant effect was found for CVL (Figure 3a and 3b). (In the placebo group, animal #14275 which displayed a controller profile was excluded from statistical analysis). In almost all tissues, a small but statistically significant decrease was observed for SIV-DNA (p = 0.03 for spleen, peripheral LN, mesenteric LN and colon) (Figure 3c). The treatment also induced a significant decrease of 2LTR SIV DNA in the spleen, the peripheral LN, and the colon (p = 0.03 for each tissue) (Figure 3d); while for SIV-RNA levels the decrease was significant (p = 0.03) only in peripheral LN (Figure 3e).

### Initiation of AZT/3TC/IDV therapy after the viremia peak results in limited and tissue-dependent effects on viral replication and dissemination

We then addressed the issue whether similar effects could be observed after maximal viral dissemination, at a time where viral reservoirs are expected to be fully established. The same treatment was therefore initiated (day 14 pi) just after viremia peak in a group of five macaques. PVL, CVL, total SIV-DNA, 2LTR SIV-DNA and SIV-RNA levels were determined 14 days later (day 28 pi) in different tissues and compared to levels of three placebo animals also euthanized at day 28 pi.

**Figure 4:**
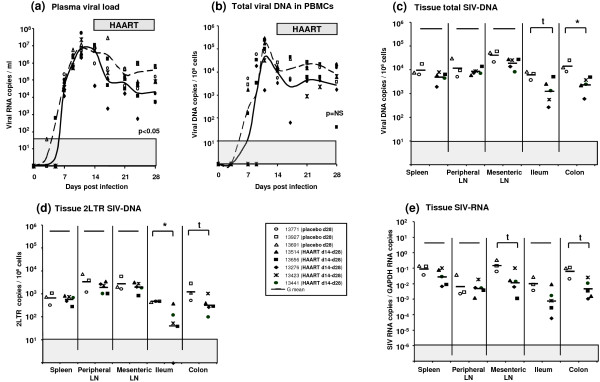
**Viral dynamics in SIV-infected macaques receiving a short-term HAART just after the viremia peak**. Among 8 animals infected since 14 days, 5 animals received the AZT/3TC/IDV combination (HAART d14-d28: black symbol) for 14 days whereas 3 animals received a placebo (placebo d28: open symbol) for the same period. All the animals were killed at the end of treatment (28 days pi). HAART initiated at 14 days pi conducted to a significant reduction of PVL (**a**) but a non significant decrease of CVL (**b**). Viral dissemination (**c**) and replication (**d,e**) were reduced at different levels depending on the tissue considered: in the gut, the decrease was significant, whereas in spleen and peripheral LN, the viral dissemination and replication were almost maintained. *: indicated a significant difference (p < 0.05) and t a trend (0.05 < p < 0.06) using a Mann-Whitney test. LN: lymph node, G mean: geometric mean. The grey area indicates the quantitative threshold of our qRT-PCR and qPCR assays.

In HAART treated animals, the effect of treatment was limited. Plasma viral load was significantly lower than in placebo macaques (p = 0.02) (Figure 4a). However, we did not observe any significant effect on CVL, total SIV-DNA, 2LTR or SIV-RNA levels, in the spleen or peripheral lymph nodes (Figure 4b-e). By contrast, the three viral markers were reduced in the digestive tract (Figure 4c-e), reminiscent of animals treated during early chronic infection.

**Figure 5:**
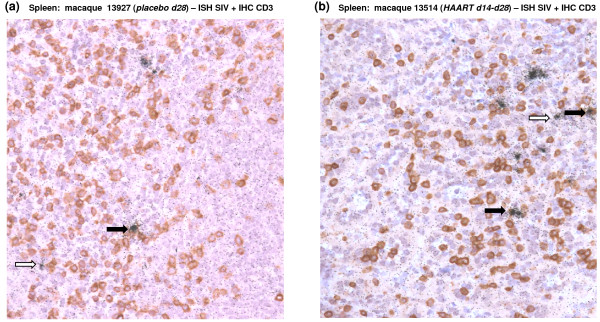
**SIV-producing cells in the spleen of SIV-infected macaques receiving a short-term HAART just after the viremia peak**. SIV-RNA detection in spleen section from a representative placebo d28 animal (#13927) (**a**) and a representative HAART d14-d28 animal (#13514) (**b**) using ^35^S in situ hybridization and T cell staining with anti-CD3 antibodies. Black arrows indicate SIV+ CD3+ cells and white arrow SIV+CD3-cells. Magnification: 200×.

As for animals treated during early chronic infection, the weak effect of HAART in spleen or peripheral LN could be the consequence of the infection of long-lived cells such as macrophages. In order to identify which cell type sustained SIV production in secondary lymphoid tissues of HAART treated macaques, we characterized SIV-RNA production using *in situ *hybridization together with T cell staining with anti-CD3 antibodies. In the spleen of placebo treated animals, SIV producing cells were mainly CD3+ (black arrow, Figure 5a). Some CD3-cells also produced weak quantities of SIV-RNA (white arrow, Figure 5a). In HAART treated animals, we confirmed the weak effect of treatment on SIV replication in the spleen with a number of SIV-RNA producing cells equivalent to placebo animals. Similar to previous observation of Cavert *et al*. in HIV infected patients [10], SIV producing cells in the spleen of macaque treated with HAART just after viremia peak were mainly T cells and not macrophages as they generally express CD3 (Figure 5b).

### 3TC concentrations vary with tissue and are inversely correlated with viral load

**Figure 6:**
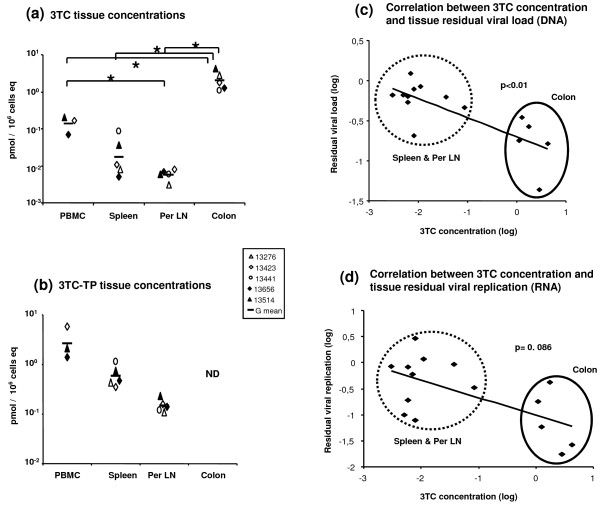
**3TC levels in tissues of SIV-infected macaques receiving a short-term HAART just after the viremia peak**. 3TC and 3TC-TP were measured in the tissues of macaque receiving the AZT/3TC/IDV combination between 14 and 28 days pi (HAART d14-d28). 3TC concentration was high in the colon, 100 times lower in the spleen and 300 times inferior in peripheral LN (**a**). 3TC-TP showed the same trend in distribution (**b**). Correlation between 3TC concentration and residual viral DNA (**c**) and RNA (**d**) in the lymphoid tissues showed an inverse relationship between 3TC concentration and the residual viral load (**c**). *: indicated a significant difference (p < 0.05) using a Mann-Whitney test. G mean: Geometric mean, Per LN: peripheral lymph node.

As the persistent viral replication in secondary lymphoid tissues did not seem to be related to infection of long-lived cells such as macrophages, we then hypothesized that it could be linked to poor antiviral activity. These could be due to either a poor diffusion of drugs in the tissue, active efflux from target cells or reduced nucleoside reverse transcriptase inhibitor (NRTI) phosphorylation by endocellular kinases. Drug pharmacokinetics (PK) can differ between species; however, we have previously shown [11] that 3TC in macaques treated with the same AZT/3TC/IDV combination displays very similar PK to humans. Thus, we first focused our measurements on the concentration of 3TC in different tissues of macaques treated with HAART between days 14 and 28 pi. Consistent with high impact of HAART on SIV replication in the gut, we found the highest 3TC concentration in the digestive tract (G mean 2 nM/10^6 ^cell equivalents (eq) in colon). The concentration in the spleen was about 100 times lower (G mean: 0.017 nM/10^6 ^cell eq), and the concentration in the peripheral LN was more than 300 times lower, than in colon (G mean: 0.006 nM/10^6 ^cell eq) (Figure 6a). Tissue cell concentrations of 3TC-TP, the active form of the drug, varied in the same way (Figure 6b); and transformation rates -- calculated by 3TC-TP concentration/3TC concentration -- were similar for each tissue tested (G means were 19.4 in PBMC, 26.2 in spleen and 25.7 in peripheral LN), indicating that efflux and phosphorylation of 3TC were not the limiting factors of antiviral activity in the different tissues. Interestingly, SIV-DNA level as indicator of residual viral load in tissues was inversely correlated (p < 0.01) with 3TC local concentration (Figure 6c). Even though the correlation did not reach statistical significance (p = 0,086), SIV-RNA level as indicator of residual viral replication also seemed to be inversely associated with 3TC local concentration (Figure 6d). We also investigated the concentrations of AZT and IDV in the same tissues. For AZT we found concentrations below the quantification threshold in almost all tissues explored (peripheral LN, spleen and colon); this may be due to the short half-life of AZT (data not shown). For IDV we found the same trend of diffusion as for 3TC with much higher concentrations in the colon (G mean: 102 pMol/10^6 ^cell eq) than in the spleen (G mean: 0,094 pMol/10^6 ^cell eq, p < 0.05). These results suggest that higher levels of residual viral replication in peripheral LN and spleen result, at least partially, from the low diffusion of antiviral drugs in these tissues.

## Discussion

We showed that a short term HAART can reduce viral dissemination and replication in all tissues when initiated very early after intravenous inoculation of SIVmac251, before the viremia peak. In animals treated just after the initial viremia peak or during early chronic infection, we observed significant differences in HAART efficacy depending on the tissue considered. Maximum inhibition occurred in the digestive tract of macaques. These results are in accordance with those described in humans showing a rapid and large decrease of viral replication in GALT after successful HAART [12-15]. Although the GALT is considered today as the major site of HIV replication, there are no data about local diffusion of antiretroviral drugs in these tissues. The dosages of 3TC and IDV we performed demonstrate, for the first time, that two commonly used antiretrovirals can diffuse very efficiently in the digestive tract, thus probably explaining the high treatment efficacy observed in this tissue. As we had recently shown [3], in the same SIV macaque model, the plasma viral load mainly reflects the viral replication in the digestive tract. We could therefore suppose that the rapid decrease in plasma viral load observed after HAART initiation is mainly due to the control of viral replication in the gut.

Despite a strong effect in the GALT, currently used antiretroviral combinations are not sufficient to eradicate the virus. We therefore assume that residual replication in pharmacological sanctuaries and/or viral reservoirs could provide an explanation to the incomplete success of therapy. In patients treated with suboptimal regimens, like the use of only two NRTI, no significant changes were observed in viral replication in LN or tonsils, even after control of PVL [16-19]. Adding a protease inhibitor (PI) increases the efficiency of treatment without achieving a full control of replication even in infected patients with long term HAART [10,17,20,21].

Our results confirm in the SIV-infected macaque model that HAART has limited impact on viral replication in secondary lymphoid tissues in spite of efficient control of PVL or replication in the gut. Contrary to Solas *et al*., who did not find any relationship between PI levels and HIV RNA levels in the tissues [22], the 3TC dosages we performed showed for the first time that low antiretroviral efficacy in spleen and peripheral lymph nodes could be related to poor drug diffusion in these organs, therefore favouring residual replication and viral persistence.

Corroborating the data from Kinman *et al*. [23], who showed very low diffusion of IDV in the lymph node of macaque after oral administration, the very low level of IDV measured in the spleen and peripheral lymph nodes of treated macaques confirm that secondary lymphoid tissues could act as real pharmacological sanctuaries.

Although one of the major limitations of our study is that drugs have been administered during a short period (14 to 28 days), several studies in humans indicate that even after several years of HAART, viral mRNA is still produced in peripheral lymph nodes. This confirms that the poor drug diffusion we observed in lymph node and spleen could provide a simple explanation to the absence of virus eradication. As suggested by Stellbrink [24], this residual replication in lymphoid tissues could also permanently seed the latent reservoir.

In the central nervous system (CNS) and the testis, the low levels of antiretroviral can be explained by poor diffusion across the blood-brain or blood-testicular barriers because of drugs efflux by ABC transporter [22,25,26]. In the peripheral lymph node mononuclear cells, we previously demonstrated a higher level of P-gp mRNA expression than in PBMC [27]. However, we did not observe here any differences in the ratios of 3TC and 3TC-TP between the different tissues, indicating that efflux from the cell or kinases activities are not the limiting factors. It is therefore unlikely that P-gp activity is involved in the poor diffusion of antiretroviral we observed in secondary lymphoid tissues.

We also demonstrated that pharmokinetics and pharmacodynamic parameters are important to consider not only in the treatment of infected patients but also in preventive approaches of HIV transmission like post-exposure prophylaxis. After intravenous inoculation of SIVmac251, infection could not be prevented even if AZT/3TC/IDV combination was initiated within a few hours, confirming our previous results [9,28]. Recently [11] we have shown that the same regimen prevents vaginal transmission of the same virus, probably because of initial viral compartmentalization and low dissemination [29] in association with good diffusion of NRTI in the female genital tract [30]. Our results thus demonstrate the need to improve antiretroviral biodistribution for better efficacy and limitation of the pharmacological sanctuaries that allow residual viral replication.

## Conclusions

When initiated before the peak of viremia, a short-term antiretroviral treatment can impact viral dissemination and replication in almost all tissues. In this case, the treatment is more effective when initiated earlier. If the identical treatment is started after the peak of viremia, or during chronic infection, the effect of short-term HAART seems to vary according to the tissue considered. In the gut, where antiretroviral drugs diffuse easily, the viral burden decreases rapidly; whereas in secondary lymphoid tissues, poor diffusion of the antiviral drug could explain the weak effect of treatment on the tissue viral load.

## Methods

### Animals, infections, treatment and tissue collection

We studied 33 young adult male cynomolgus macaques (*Macaca fascicularis*), each weighing between 2.7 and 4.5 kg. Studies were conducted in accordance with European guidelines for animal care and all experiments were approved by the ethics committee for animal experimentation "Ile de France Sud" (Paris, France). All macaques were inoculated intravenously with 50 AID_50 _of pathogenic SIVmac251 and divided into nine groups.

Six animals were treated with AZT (4.5 mg/kg) and 3TC (2.5 mg/kg) subcutaneously twice daily and indinavir (60 mg/kg), orally, twice daily. The treatment was initiated after viral set point (day 150 pi) and the animals were killed after 14 days (chronic HAART 14 d group) and 28 days (chronic HAART 28 d group) of treatment. In parallel, 3 untreated animals were also killed at 150 days pi (chronic untreated).

Four other animals received the AZT/3TC/IDV combination as early as 4 h post-infection and continued until day 14 when the animals were killed (HAART 4h-d14 group). Four animals receiving a placebo in the same conditions were also killed at 14 days pi (placebo d14).

A group of four animals received the same HAART between days 7 and 21 pi (HAART d7-d21 group), then the animals were killed on day 21 pi. Four animals receiving a placebo in the same conditions were killed at 21 days pi (placebo d21).

In last group, 5 animals received the HAART treatment between day 14 and day 28 pi and were killed on day 28 (HAART d14-d28 group). Three animals receiving a placebo in the same conditions were killed at the same time (placebo d28).

Immediately after killing the animals, tissue samples from the spleen, peripheral lymph nodes (inguinal or axillary) mesenteric lymph nodes, ileum and colon were collected in quadruplicate and stored at -80°C. In order to reduce the heterogeneous presence of lymphoid tissue in the gut, we collected and processed large samples for ileum and colon (250 to 400 mg).

### Virological measurements in the blood

Plasma and cell-associated viral loads were determined as previously described [9,31].

### Virological measurements in the tissues

RNA and DNA extraction as well as quantification of total SIV DNA, SIV 2 LTR circles and SIV RNA in tissue were performed as previously described [3].

### *In situ *hybridisation

SIV gag *in situ *hybridization combined with immunohistochemistry for T cell markers was performed as previously described [32]. The specificity of the hybridization signal was systematically checked by hybridizing sense probes on successive sections. Image acquisition and analysis were performed on a Nikon i90 photomicroscope using NIS-elements software.

### Determination of antiretroviral concentration in tissues

3TC, 3TC-TP, AZT and IDV were assayed by liquid chromatography coupled with tandem mass spectrometry (LC-MS/MS) or a modification of these methods, as previously described [33-35].

### Statistical analysis

Statistical analyses were carried out using Stat View software (SAS institute Inc, Cary, North Carolina, USA). Plasma and cell-associated viral load as well as SIV-RNA, total SIV-DNA and 2LTR SIV-DNA were compared in placebo and HAART-treated macaques using a nonparametric Mann-Whitney test. Differences in 3TC and IDV concentration in lymphoid tissues were assessed by the same test. The correlation between 3TC concentration and residual viral replication/load in tissue, were evaluated using a nonparametric Spearman correlation test.

## Competing interests

The authors declare that they have no competing interests.

## Authors' contributions

RLG, OB and PR conceived and designed the experiments; OB, PS, AM, CR, LDG, RLG and PR performed the experiments; OB, PS, AM, CR, NDB, LDG, HB, PR and RLG analyzed the data; HB contributed reagents/materials/analysis tools; OB, RLG, PS, PR and AM wrote the paper. All authors read and approved the final manuscript.
